# MRI-Based Optimization Design of the Pre-Spinal Route of Contralateral C7 Nerve Transfer for Spastic Arm Paralysis

**DOI:** 10.3389/fsurg.2022.837872

**Published:** 2022-06-29

**Authors:** Hua-Li Zhao, Yun Gao, Ai-Ping Yu, Yi-Min Wei, Yun-Dong Shen, Su Jiang, Yan-Qun Qiu, Jing Yu, Zong-Hui Liang

**Affiliations:** ^1^ Department of Radiology, Jing’an District Central Hospital, Shanghai, China; ^2^ Department of Hand and Upper Extremity Surgery, Jing’an District Central Hospital, Shanghai, China; ^3^ Department of Hand Surgery, Huashan Hospital, Shanghai Medical College, Fudan University, Shanghai, China

**Keywords:** magnetic resonance imaging (MRI), spastic arm paralysis, contralateral C7 transfer, pre-spinal routes, optimization design

## Abstract

**Purpose:**

The prespinal route of contralateral cervical 7 nerve transfer developed by Prof. Wendong Xu helps realize the direct anastomosis of the bilateral cervical 7 nerves. However, 20% of operations still require a nerve graft, which leads to an unfavorable prognosis. This study aims to explore the optimized prespinal route with MRI to further improve the prognosis.

**Methods:**

The current study enrolled 30 patients who suffered from central spastic paralysis of an upper limb and who underwent contralateral cervical 7 nerve transfer via Prof. Xu’s prespinal route through the anterior edge of the contralateral longus colli. MRI images were used to analyze the route length, vertebral artery exposure, and contralateral cervical 7 nerve included angle. Three prespinal routes were virtually designed and analyzed. The selected optimal route was applied to another 50 patients with central spastic paralysis of an upper limb for contralateral cervical 7 nerve transfer.

**Results:**

By the interventions on the 30 patients, the middle and posterior routes were shorter than the anterior route in length, but with no statistical difference between the two routes. Of 30 contralateral vertebral arteries, 26 were located at the posterior medial edge of the longus colli. The average included angles of the anterior, middle, and posterior routes were 108.02 ± 7.89°, 95.51 ± 6.52°, and 72.48 ± 4.65°, respectively. According to these data, the middle route was optimally applied to 50 patients, in whom the rate of nerve transplantation was only 4%, and no serious complications such as vertebral artery or brachial plexus injury occurred.

**Conclusion:**

The low rate of nerve transplantation in 50 patients and the absence of any serious complications in these cases suggests that the middle route is the optimal one.

## Introduction

Brachial plexus injury caused by trauma, birth injury, and other things can seriously impact the function of the paralyzed upper limb, and spastic hemiplegia of the upper extremity due to central paralysis is a common sequela that significantly affects the quality of life of patients (1–3). The number of such patients is very high, which can have practical consequences for those caring for them and for society as a whole. The effectiveness of common rehabilitation therapy is extremely limited, which makes it difficult to improve the function of the paralysed limb. The prespinal route of “contralateral cervical 7 (C7) nerve transfer,” which is the route through the anterior edge of the contralateral longus colli, the anterior edge of the vertebral body, the posterior edge of the esophagus, and the anterior edge of the longus colli on the paralyzed side, hence known as the anterior route, was developed by Prof. Wendong Xu’s team and has become a new and effective approach to the treatment of this condition (4, 5).

During the operation, if the bilateral C7 nerves cannot be directly anastomosed, other nerves need to be grafted, which will reduce the surgical effect. Compared with the early anterior cervical subcutaneous path, Prof. Xu’s prespinal route significantly shortens the distance of transposition and achieves direct anastomosis of bilateral C7 nerves, thus reducing trauma, the length of the operation, recovery time, and time for nerve regeneration, thus improving the therapeutic effect. However, 20% of procedures still necessitate the use of a graft regardless of the shortened pathway (6). In addition, the regional anatomy of the transposition path is relatively complex, mainly involving the vertebral artery and brachial plexus, which is closely related to the prespinal route (7). All these factors can restrict the wide application of the operation.

At present, imaging examination has become an important technique in preoperative evaluation, since it is capable of clearly showing the location relationship where the anatomical structures are located in the surgical area, performing accurate data measurement and precise positioning, and providing a data basis for a particular surgical intervention. For the prespinal route of contralateral C7 nerve transposition, however, a standard preoperative evaluation system is not available. Therefore, the objective of our study was to explore the optimized prespinal route by MRI simulation and evaluate the position and relationship between the locations of important anatomical structures, so that we could shorten the route distance, evaluate the safety of the surrounding structures, improve the curative effect, and avoid serious complications. This could thus provide a scientific basis for clinically designing a shorter and safer transfer route for direct and tension-free anastomosis of the bilateral C7 nerve.

## Materials and Methods

### Patients

A total of 80 CNS injury patients were enrolled in this study, 30 of whom underwent contralateral C7 nerve transfer surgery via the prespinal route through the anterior edge of the contralateral longus colli from June 2018 to December 2019, and the remaining 50 of whom had received the same surgery via the prespinal route through the middle of the contralateral longus colli from January 2020 to December 2020. There were no statistically significant differences in age and gender at scanning within the groups that were to receive brachial plexus MRI examination. Prior to the examination, all patients signed the informed consent form for the current study, the protocol of which was approved by the Research Ethics Committee of Shanghai Jingan District Central Hospital.

### Surgical Procedure

**Figure 1:**
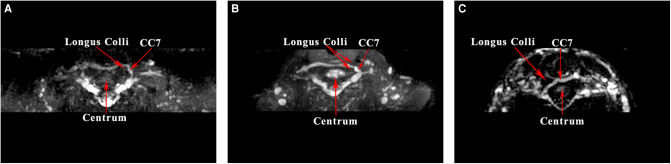
contralateral cervical 7 (CC7) nerve transfer operation on MRI by 3D CUBE-STIR sequences: (**A**) anterior route, (**B**) middle route, (**C**) posterior route.

As previously reported (6), the surgical procedure was as follows: A 15  cm transverse incision was made approximately 2  cm superior to the clavicle at the bottom of the neck so that the brachial plexus nerves were bilaterally exposed, superior to the clavicle. On the paralyzed side, the C7 nerve was severed near the intervertebral foramen, and on the nonparalyzed side, the C7 nerve was severed, as distally as possible, proximal to the point at which it combined with the fibers of other brachial plexus nerves. The anterolateral aspect of the C7 vertebral body was bluntly dissected so that the esophagus was exposed anterior to the vertebral body, thus creating a conduit between the spinal column and the esophagus. Afterward, the cut end of the C7 nerve on the nonparalyzed side was drawn through the prespinal route (the anterior, middle, or posterior route) to the paralyzed side to be anastomosed, directly without a graft or indirectly with a graft, to the cut end of the C7 nerve using microsurgical epineurium suturing (Figure 1). The whole procedure was performed by a senior surgeon.

### MRI Scanning

MRI scanning was performed using a 3.0  T scanner on the patient, who lay supine, with their arm positioned by their side. The standard sequence protocol involved axial T1WI, axial T2WI, 3D-fast imaging employing steady-state acquisition-constructive interference steady state, and 3D fast spin echo with an extended echo train acquisition (CUBE)-short tau inversion recovery (STIR). The scanning parameters of the T1WI sequence were as follows: FOV 240  mm, thickness/interval 3/0  mm, resolution 288 × 256, TR 582  ms, and TE 6.7  ms and those of the 3D CUBE-STIR sequence were as follows: FOV 280  mm, thickness/interval 1.6/0  mm, resolution 320 × 224, TR 4,750  ms, and TE 179  ms. The brachial plexus MRI images of each patient were carefully evaluated.

The MRI datasets were imported into the post-processing software package of Image J 1.52p (Wayne Rasband National Institute of Health, USA). On T1WI images at the level of the C7 nerve outlet of the intervertebral foramen, the following were visible: the trace lines from the contralateral to the paralyzed side along the anterior edge of longus colli on the contralateral side, the anterior edge of the vertebral body, the posterior edge of the esophagus, the anterior edge of the longus colli on the paralyzed side (the anterior route); through the middle of longus colli on the contralateral side, the anterior edge of the vertebral body, the posterior edge of the esophagus, the anterior edge of the longus colli on the paralyzed side (the middle route); and the posterior edge of longus colli on the contralateral side, the anterior edge of the vertebral body, the posterior edge of the esophagus, and the anterior edge of the longus colli on the paralyzed side (the posterior route). We usedthe segmented line tool to draw different surgical paths manually before employing the fit spline to smooth the path curve and the trajectory measurement tool to automatically calculate the lengths of different surgical paths.

**Figure 2:**
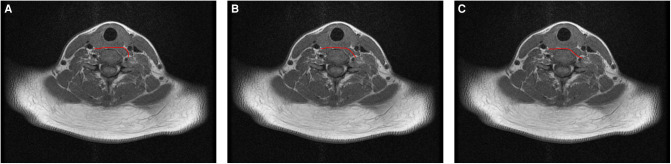
Prespinal routes of CC7 transfer surgery on T1-weighted images (T1WI): (**A**) anterior route, (**B**) middle route, (**C**) posterior route.

**Figure 3:**
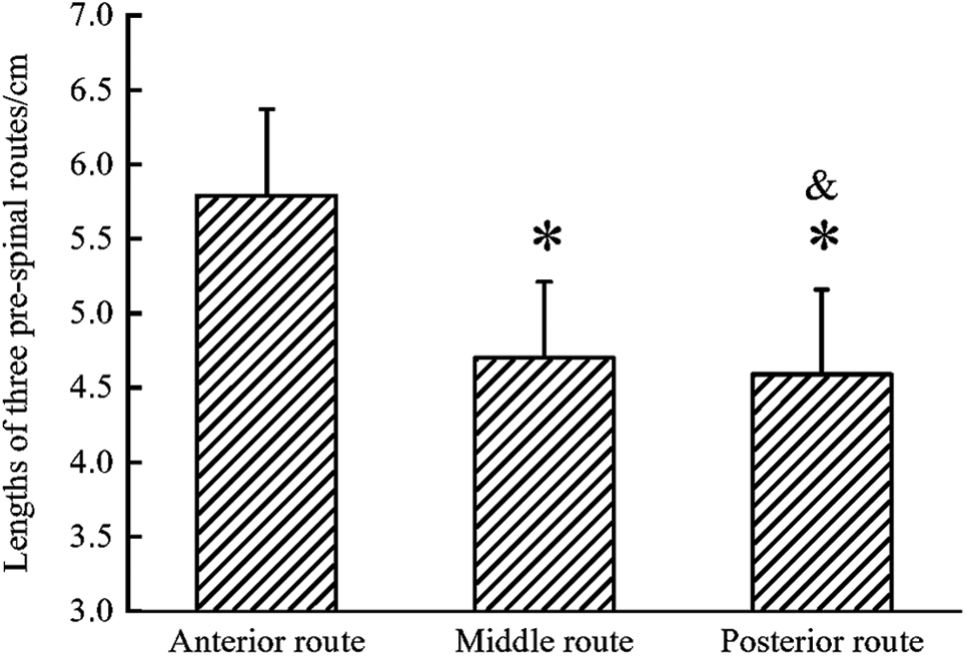
Statistical chart of lengths of three prespinal routes on T1WI. The lengths of the two were statistically shorter than that of the anterior route (the middle, *p* < 0.001; the posterior, *p* < 0.001). No significant difference was observed between the middle and the posterior route (*p* = 0.57).

**Figure 4:**
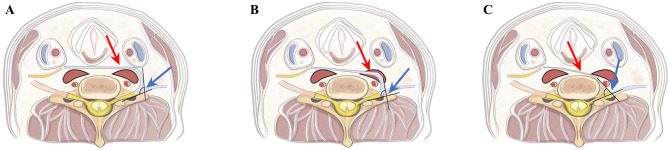
The included angle of CC7 nerve transfer via three prespinal routes: (**A**) anterior route, (**B**) middle route, (**C**) posterior route.

**Figure 5:**
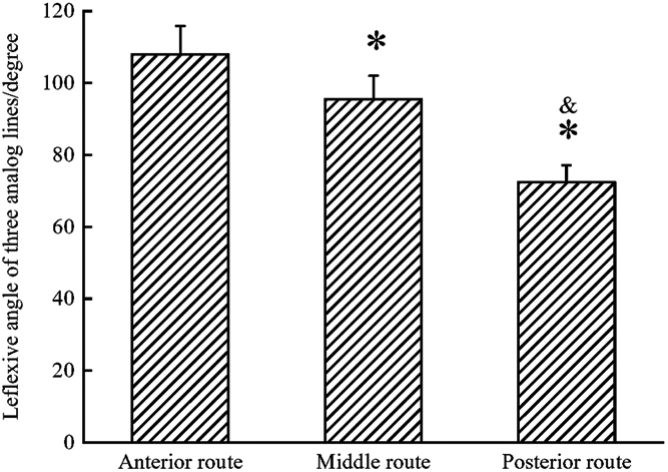
Statistical chart of the CC7 nerve included angles of three prespinal routes. *The included angles of the two were significantly smaller than that of the anterior route (the middle, *p* < 0.001; the posterior, *p* < 0.001). ^&^The included angle of the middle route was larger than that of the posterior route (*p*<0.001).

In all the patients, the lengths of the anterior, middle, and posterior routes were automatically measured using Image J software (Table 1; Figures 2, 3), as were the contralateral C7 nerve included angles of the three prespinal routes (anterior, middle, or posterior) simulated at the level of the superior margin of the C7 vertebra on the preoperative T1WI sequence (Table 2; Figures 4, 5).

**Table 1:** The lengths of three prespinal routes on T1WI.

Group	*N*	Mean (cm)	Std. deviation	Std. error	95% Confidence interval	
					Lower bound	Upper bound
Anterior route	30	5.79	.59	.06	5.66	5.91
Middle route	30	4.70*	.51	.05	4.60	4.81
Posterior route	30	4.59*	.57	.06	4.47	4.71

*T*1*WI, T*1 *weighted imaging;* *p < 0.001; ^&^*p* > 0.05.

**Table 2:** Included angle of the CC7 nerve via three prespinal routes.

Group	*N*	Mean (∠)	Std. deviation	Std. error	95% Confidence interval	
					Lower bound	Upper bound
Anterior route	30	108.02	7.89	1.76	104.33	111.72
Middle route	30	95.51*	6.52	1.46	92.46	98.56
Posterior route	30	72.48*^&^	4.65	1.04	70.30	74.65

*CC*7*,* *contralateral cervical* 7; *p < 0.001; ^&^*p* > 0.05.

### Statistical Analysis

The Kolmogorov–Smirnov test was applied to each continuous variable to examine whether a normal distribution could be assumed, after which the variable was summarized as mean ± SD as appropriate. The group means were compared using one-way ANOVA or independent sample *t*-tests, and the results were presented as odds ratios with 95% confidence intervals. A value of *p* < 0.05 was considered statistically significant. The statistical analysis was performed on SPSS, version 17.0 (SPSS, Chicago, IL, USA).

## Results

In the contralateral C7 nerve transfer operation, the three prespinal routes were presented on MRI by using the 3D CUBE-STIR sequence (Figure 1). According to the measured lengths of the three prespinal routes simulated at the level of the superior margin of the C7 vertebra on the preoperative T1WI sequence, the average lengths of the anterior, middle, and posterior routes were 5.79 ± 0.59  cm, 4.70 ± 0.51  cm, and 4.59 ± 0.57  cm, respectively. Statistical analysis showed no significant difference between the middle and the posterior route (*p* = 0.57), while the anterior route was significantly longer than the other two (all *p* < 0.001; Table 1; Figures 2, 3).

From the measurement of the contralateral C7 nerve included angles of the three prespinal routes (the anterior, middle, or posterior route) simulated at the level of the superior margin of the C7 vertebra on the preoperative T1WI sequence (represented by a schematic map), the average included angles of the anterior, middle, and posterior routes were 108.02 ± 7.89°, 95.51 ± 6.52°, and 72.48 ± 4.65°, respectively. Statistical analysis showed significant differences between the anterior, middle, and posterior routes (all *p* < 0.001; Table 2; Figures 4, 5).

**Figure 6:**
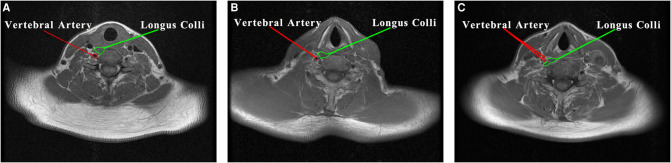
Anatomic relationship between vertebral artery and longus colli: (**A**) the vertebral artery located at the posterior medial margin of the longus colli (26/30), (**B**) the vertebral artery located at the lateral margin of the longus colli (3/30), (**C**) the vertebral artery located at the anterior margin of the longus colli (1/30).

An MRI showed that the contralateral vertebral artery was located at the posterior medial edge of the longus colli in 26 cases (26/30), at the lateral edge of the longus colli in 3 cases (3/30), and at the anterior margin of the longus colli in 1 case (1/30; Figure 6).

According to the data of the first 30 patients, who underwent contralateral C7 nerve transfer surgery through the anterior route, and of another 50 patients who underwent contralateral C7 nerve transfer surgery through the middle route, 2 were found to have nerve transplantation, the rate of which was 4%, with no occurrence of serious complications such as vertebral artery and brachial plexus injury.

## Discussion

Peripheral nerve transfer, first carried out by Prof. Yudong Gu in 1970, was first applied to patients with brachial plexus avulsion through phrenic nerve transplantation (8, 9). In 1986, academician Yudong Gu pioneered the contralateral C7 nerve transfer in the world to treat patients with total brachial plexus injury by creating the classic anterior cervical subcutaneous route contralateral C7 nerve transfer, which has been widely used (10–12). Since then, Prof. Wendong Xu has improved the surgery by developing the anterior route, for the contralateral C7 nerve transfer in treating patients with brachial plexus injury and central hemiplegia (13, 14). This approach significantly shortens the route, actualizing the direct anastomosis of the bilateral C7 nerve, reducing the rate of nerve transplantation, and achieving remarkable efficacy (4).

In our previously reported clinical study, however, we had found that the nerve transplantation rate of the anterior route in the treatment of unilateral arm paralysis due to central paralysis was 20% (6). According to the clinical follow-ups, the curative effect on the patients requiring nerve transplantation was found to be worse than that of bilateral C7 nerve direct anastomosis, increasing the difficulty of the surgery and the uncertainty of the prognosis (15). As for surgical complications, so far no serious adverse events related to the surgery have been reported in the clinical research by Prof. Xu’s team (16). However, it was reported that the total incidence of complications was 5.4% (23 of 425), which was mainly related to the prespinal route (the anterior route), exploration and transection of contralateral C7 nerve, and that two cases of more serious vertebral artery injury accounted for 0.47% and four cases of contralateral brachial plexus injury, 0.94% (7).

Therefore, we tried to use MRI images to simulate the prespinal routes to explore a better and shorter surgical path through which to reduce the probability of nerve transplantation and the risk of surgery.

On the basis of Prof. Xu’s modified prespinal route (the anterior route) (4, 5), we simulated three prespinal routes at the upper edge of the C7 vertebral body on MRI images: the anterior, middle, and posterior routes. Of the three routes, in the 30 patients who underwent contralateral C7 nerve transfer surgery through the anterior route, the anterior route was the longest, with an average length of 5.79 ± 0.59  cm. In our previously reported clinical study (6), six patients who underwent contralateral C7 nerve transfer surgery through the anterior route received nerve transplantation, and the mean length of the contralateral C7 nerve measured during the surgery (5.7 ± 0.6  cm in the anterior division and 5.2 ± 0.6  cm in the posterior division) was shorter than the anterior route (5.79 ± 0.59  cm) but longer than the middle route (4.70 ± 0.51  cm) and the posterior route (4.59 ± 0.57  cm). If the six patients received the middle route or the posterior route, therefore, a direct and tension-free anastomosis of the bilateral C7 nerve could be realized. According to the statistics of the anatomic position of the vertebral artery in the surgical access area, it was found that at the level of the superior edge of the C7 vertebral body, the vertebral artery was closely related to the longus colli, and most of the vertebral arteries located in the posterior or posterolateral part of the longus colli (17, 18). In our study, the contralateral vertebral arteries were located at the posterior medial margin of the longus colli in 26 of 30 patients, at the lateral margin of the longus colli in 3 of 30 cases, and at the anterior margin of the longus colli in 1 of 30 cases. This suggests that the vertebral artery can be more successfully avoided by taking the anterior and middle routes than by taking the posterior route.

There exist cervical thoracic ganglia in the space between the anterior part of the vertebral body and the medial margin of the longus colli at the upper edge of the C7 vertebral body (19). If the posterior route is selected, it is likely to cause sympathetic nerve and vertebral artery damage (20, 21). As realized by surgeons, when the incision of the posterior route is deeper, surgery becomes more difficult, and when the included angle of the contralateral C7 nerve is smaller in the posterior route, the C7 nerve conduction is more likely to be affected. Based on the analytical data on the three routes and where the important anatomical structures are located along the route, it could be concluded that the middle route was surgically the best, which was characterized by a short distance, high safety, and less impact on the function of the contralateral C7 nerve.

Of the 50 patients with spastic upper limb paralysis caused by central palsy who were selected to undergo the contralateral C7 nerve transfer through the middle route, only 2 patients received nerve transplantation at a rate of 4%; the rate was significantly lower when compared with that in the case of the anterior route (6/30, 20% vs. 2/50, 4%; *p* = 0.021) (6), even without structural injuries of the vertebral artery, brachial plexus, and other important tissue.

Technically mature MRI on brachial plexus is both noninvasive and safe. The simulation and optimization of the prespinal route through the anterior route and the presentation of where the anatomical structures are located in the area to be operated on facilitate the obtainment of data regarding the displacement route, the location of the adjacent important anatomical structures, the adjacent relationship, and the anatomical variation prior to the surgery. Thus, the technique provides a scientific basis for designing a better transfer route for a direct and tension-free anastomosis of the bilateral C7 nerve, avoiding the occurrence of serious complications, reducing surgical trauma, improving surgical safety and efficacy, and realizing precise and personalized treatment. As a result of this study, we can conclude that MRI can provide technical support for the popularization and application of contralateral C7 nerve transfer.

## Data Availability

The original contributions presented in the study are included in the article/Supplementary Material, further inquiries can be directed to the corresponding author.
